# Supplementary material for: Molecular Docking-Based Screening for Novel Inhibitors of the Human Immunodeficiency Virus Type 1 Protease that Effectively Reduce the Viral Replication in Human Cells
Source: J AIDS Clin Res. Author manuscript; Available in PMC 2021 Dec 22. (PMC8694022)
Supplement: Appendix [file NIHMS1713767-supplement-Appendix.pdf]

Appendix

```
AD      PQITLWQRPIVTIKIGGQLKEALLDTGADDTVLEDMNLPGRWKPKMIGGIGGFIKVRQYD 60
AD02    PQITLWQRPIVTIKVGGQLKEALLDTGADDTVLQEINLTGRWKPKMIGGIGGFAKVREYD 60

AD      QILIEICGHKAIGTVLVGPTPVNIIGRNLLTQLGCTLNF 99
AD02    QVPIEICGHKAIGTVLVGPTPANIIGRNLLTQIGCTLNF 99
```

Figure S1. Amino acid alignment of the HIV-1_{AD} and HIV-1_{AD02} PRs. Amino acid alignment based on Clustal Omega of the wild-type and (AD) and drug-resistant (AD02) PRs. Resistant residues that differ between HIV-1_{AD} and HIV-1_{AD02} are shown in red.

18 PIS IC_{50}: 62.7 PIR IC_{50}: 68.5

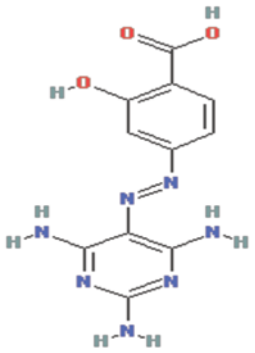

22 PIS IC_{50}: 53.5 PIR IC_{50}: 71.9

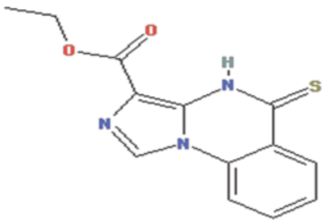

27 PIS IC_{50}: 26.0 PIR IC_{50}: 27.1

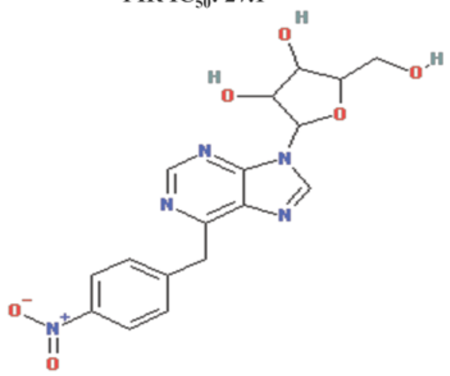

32 PIS IC_{50}: 3.8 PIR IC_{50}: 13.7

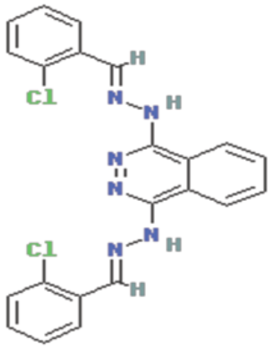

35 PIS IC_{50}: 1.43 PIR IC_{50}: 6.3

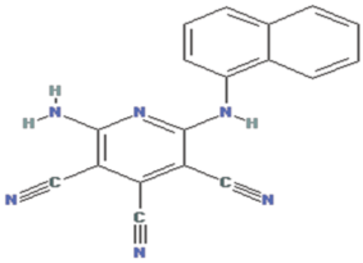

Figure S2. Chemical structure of the compounds. Chemical structure of the five lead compounds 18, 22, 27, 32 and 35. Next the identification number of each compound the IC_{50} against PI-sensitive [PIS IC_{50}] or against multi PI-resistant HIV-1 variant [PIR IC_{50}] are given in µM range.

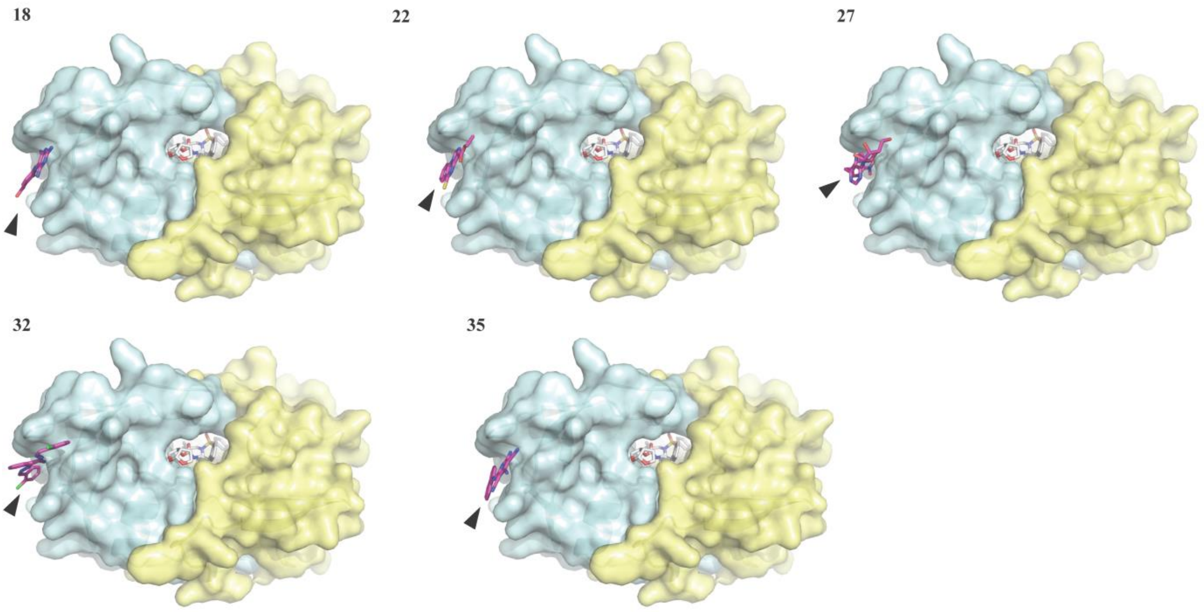

Figure S3. HIV PR subtype B wild-type in complex with compounds 18, 22, 27, 32 and 35. Computer modeling of compounds 18, 22, 27, 32 and 35 binding in the hinge region of the crystal structure of the PR homodimer complexed with inhibitor Darunavir in the active-site. Computer modeling of compounds, indicated by a black arrow, bound in the hinge region of the crystal structure of the PR homodimer complexed with PI inhibitor in the active-site (1t3r).

Table S1. Toxicity of compounds in PBMC and TZM-bl cells. NCI: database number; ID: compound identification number; toxicity is expressed relative to viability of cells in DMSO: light grey, ≤10% or black, >10%.([1]) Concentrations are expressed in μM.

**Compounds**		**Cellular Toxicity in Presence of Compounds [μM]**							
**ID**		**TZM-bl cells**				**PBMC**			
NCI	ID	25[1]	50	100	200	10	25	50	200
159456	1								
636983	8								
117285	18								
60044	19								
649152	22								

295274	27								
103650	32								
118210	34								
663619	35								
84120	36								
107192	37								
12994	39								

Table S2. Fold change of compounds (18, 22, 27, 32 or 35) alone or in combination with 25 µM RTV compared to 25 µM RTV. P values are reported in parenthesis with Bonferroni correction with significance p<0.0055; fold change >1 indicates antiviral-activity less effective (viral replication higher) than 25 µM RTV alone and significant ones are highlighted in green; fold change <1 indicates antiviral-activity more effective (viral replication lower) than 25 µM RTV alone and significant ones are highlighted in yellow; (-) not performed. ([1]) Anti-viral activity against HIV-1; ([2]) Compounds identification number.

**Concentrations of Compounds**		**Fold change of compounds compared to 25 µM RTV as reference**									
		**HIV-1$_{AD}$[1]**					**HIV-1$_{AD02}$[1]**				
		**18[2]**	**22**	**27**	**32**	**35**	**18**	**22**	**27**	**32**	**35**
**Compounds alone**	1 µM	-	-	-	5.84 (<.0001)	-	-	-	-	6.86 (<.0001)	-
	2.5 µM	-	-	-	7.14 (<.0001)	5.51 (<.0001)	-	-	-	6.85 (<.0001)	3.39 (<.0001)
	10 µM	-	-	-	4.44 (0.0597)	6.18 (<.0001)	-	-	-	2.51 (<.0001)	6.18 (<.0001)
	25	1.12	1.48	1.91	-	5.90	3.42	3.77	3.02	-	5.90

	μM	(0.2014)	(0.0127)	(<.0001)		(<.0001)	(<.0001)	(<.0001)	(<.0001)		(<.0001)
	50 μM	1.13 (0.0742)	1.37 (<.0001)	1.90 (<.0001)	-	-	3.24 (<.0001)	3.03 (<.0001)	2.18 (<.0001)	-	-
	100 uM	0.87 (0.0133)	0.52 (0.1975)	1.16 (0.7856)	-	-	2.41 (<.0001)	2.18 (<.0001)	0.81 (0.1363)	-	-
**Compounds with 25 μM RTV**	1 μM	-	-	-	2.59 0.0279	-	-	-	-	1.60 (<.0001)	-
	2.5 μM	-	-	-	2.18 (0.2595)	2.40 (0.3996)	-	-	-	1.12 (0.3157)	2.40 (0.0150)
	10 μM	-	-	-	0.71 (0.0473)	2.84 (0.8317)	-	-	-	0.37 (<.0001)	2.84 0.0289
	25 μM	0.54 (1.1162)	0.51 (0.1369)	0.53 (0.0286)	-	0.41 (0.0011)	0.75 (0.0206)	1.18 (0.1558)	0.53 (0.0044)	-	0.41 (<.0001)
	50 μM	0.32 (1.1273)	0.29 (0.0021)	0.32 (0.0026)	-	-	0.60 0.0010	0.57 (<.0001)	0.34 (<.0001)	-	-
	100 μM	0.40 (0.8741)	0.17 (0.0011)	0.13 (0.0011)	-	-	0.53 (<.0001)	0.63 (<.0001)	0.35 (<.0001)	-	-
